# Physical Literacy through Active plaY and environmental affordances in outdoor kindergarten settings: a multidimensional approach to Unveiling Potential (PLAY UP) – study protocol for a cluster-randomised trial

**DOI:** 10.1136/bmjopen-2026-120976

**Published:** 2026-07-01

**Authors:** Gabriela Almeida, Bruno Gonçalves, Carlos Luz, Luís Galhardas, Helen Bilton, José Marmeleira

**Affiliations:** 1 Department of Sport and Health, University of Évora, School of Health and Human Development, Evora, Portugal; 2 Comprehensive Health Research Centre (CHRC), Universidade de Évora, Evora, Portugal; 3 Escola Superior de Educação de Lisboa, Ci&DEI, Instituto Politécnico de Lisboa, Lisbon, Portugal; 4 Institute of Education, University of Reading, Reading, UK

**Keywords:** child education, health, intervention, physical fitness

## Abstract

**Introduction:**

Outdoor active play is essential for children’s health, well-being and holistic development. However, there is a lack of high-quality evidence on effective sustainable and scalable interventions that leverage outdoor play and environmental affordances to promote physical literacy, including motor competence, physical activity, engagement and motivation for movement in early childhood. In particular, limited research has examined how outdoor kindergarten environments can be intentionally used and optimised to support diverse forms of active play through social and other potential affordances, nor how educators can be supported to facilitate such processes. The PLAY UP project aims to address this gap by investigating how a co-created, affordance-based intervention, developed in collaboration with children and supported by educator training, can enhance preschool children’s engagement in active play and multidimensional development in outdoor kindergarten settings.

**Methods and analysis:**

This study describes the protocol of a cluster-randomised trial to be conducted in Portugal, involving three kindergartens and up to 215 preschool children, their educators and parents. The protocol includes an initial pilot phase (approximately 75 children from one kindergarten) to assess feasibility, acceptability and implementation procedures, followed by a main study phase with intervention and wait-list control clusters. The intervention comprises 15 sessions delivered in outdoor kindergarten settings. PLAY UP adopts a multidimensional and multimethod approach combining quantitative and qualitative methods. Outcomes related to physical literacy, including physical activity, motor competence, engagement, enjoyment and motivation for movement, are assessed using questionnaires, standardised tasks, accelerometry, Global Positioning System-based positioning technologies and systematic observations, complemented by interviews. Data will be analysed using cluster-aware statistical models and integrative mixed-methods approaches.

**Ethics and dissemination:**

This study has been approved by the Ethics Committee of the University of Évora (Ref. #24101). Written informed consent was obtained from parents or legal guardians, and all data will be handled confidentially in accordance with ethical and data protection standards. Study findings will be disseminated through open-access peer-reviewed publications, scientific conferences and knowledge-transfer activities targeting education professionals and relevant stakeholders.

**Trial registration number:**

NCT07355179.

**STRENGTHS AND LIMITATIONS OF THIS STUDY**

- Cluster-randomised trial design conducted in real-world kindergarten settings, enhancing ecological validity and relevance for early childhood education practice.
- Affordance-based, co-created intervention, developed with children and supported by educator training, addressing both environmental and pedagogical determinants of physical literacy.
- The limited number of clusters may constrain statistical power and generalisability at the cluster level, despite an adequate individual-level sample size.
- The context-specific nature of outdoor kindergarten environments may limit transferability to settings with different spatial, cultural or pedagogical characteristics.
- Outdoor-based physical activity assessments may be influenced by seasonal and weather-related variability, potentially affecting interpretation of movement-related outcomes.

## Introduction

Physical literacy (PL) has been conceptualised as a holistic and multidimensional construct that extends beyond the development of motor skills alone. It encompasses physical competence, physical activity (PA), motivation, confidence, as well as knowledge and understanding, enabling individuals to engage in PA throughout the lifespan.^1 2^ In addition to these dimensions, PL integrates affective and cognitive components related to PA, reinforcing its comprehensive nature.^3^ From a theoretical perspective, PL is grounded in embodied and relational approaches, which conceptualise movement as central to how individuals interact with and make sense of their environment.^4^ In this sense, physically literate children develop motor competence and acquire diverse movement experiences through engagement with a variety of environments (eg, land, snow or ice, water or air), contexts (eg, play, movement or sport experiences), and social agents (eg, educators, peers).^5^ To foster PL, children need to engage in varied and enjoyable PA that promotes positive emotions, meaningful experiences, sustained motivation for movement and a meaningful connection with their environment^6^.

In early childhood, a critical period for development, meaningful movement experiences and supportive pedagogical environments are essential for fostering PL^7^. In this context, active play emerges as a key means through which children develop physical, cognitive and socioemotional competencies. In kindergarten settings, outdoor active play refers to spontaneous, unstructured and often vigorous movement involving gross motor or whole-body activity in which young children freely choose enjoyable ways to expend energy^8^; it has been identified as a particularly powerful approach to fostering PL while supporting motor, socioemotional and cognitive development,^9^ engagement in outdoor play has been shown to support motor skills development,^10^ executive functioning,^11^ promote social behaviour and peer interactions^12^ and contribute to overall well-being, learning and holistic development.^13^ The effectiveness of outdoor active play is closely linked to the characteristics of the environment in which it occurs. Kindergarten outdoor spaces play a crucial role in fostering PL, PA, play, peer interaction and meaningful engagement with the surrounding environment.^14–16^ These environments are inherently rich in affordances, defined as possibilities for action^17^, which support exploration, interaction and movement, thereby contributing to children’s cognitive, linguistic, socioemotional and physical development.^13 18^ A physically literate child is able to interpret the environment and identify affordances,^19^ including both physical and social affordances, where objects, materials, spaces and peers provide opportunities for interaction.^20^ The characteristics of these affordances play a key role in shaping children’s PA behaviours and social engagement,^21^ while specific features of outdoor environments create opportunities for active play and interaction.^22^ Through engagement with outdoor environments, children explore and actualise affordances,^14^ progressively develop motor competence and body control^18 23^ and enhance self-regulatory and relational skills.^13 24^

Beyond developmental outcomes, outdoor environments also provide important physiological and health benefits. Exposure to natural elements such as sunlight and fresh air promotes PA and contributes positively to health, well-being, learning and development.^13 25^ Outdoor play supports vitamin D synthesis and serotonin regulation, contributing to immune function and mental health,^13^ while environmental variability enhances sensory integration and musculoskeletal development.^26^ Additionally, active outdoor play has been associated with more favourable movement behaviour patterns, including higher levels of PA and reduced sedentary time,^27^ as well as a reduced risk of myopia and its progression in children.^28^ Recent evidence further highlights the broad benefits of outdoor experiences in early childhood education settings, including improvements in behavioural regulation, learning outcomes, executive functioning, social and emotional skills and attention-related behaviours.^29^ Moreover, greater frequency of outdoor play has been associated with enhanced emotional intelligence and socioemotional development.^30^

Despite these benefits, children’s opportunities for active play have declined, increasingly replaced by sedentary indoor activities. Consequently, prolonged time spent in indoor environments raises concerns about air quality, as kindergarten settings often contain chemical pollutants that may impact children’s neurodevelopment and overall health.^31^ In Portugal, this decline is particularly alarming, raising growing concerns. Schools, which should serve as pivotal settings for encouraging outdoor active play, are no longer actively promoting outdoor activities.^32^ This is particularly alarming given that Portuguese preschoolers spend approximately 40 hours per week in kindergarten,^33^ where they should have ample opportunities to engage in outdoor PA and active play. This period is also critical for motor development, as children are expected to reach the mature stage of fundamental movement skills by around 7 years of age, highlighting the central role of early childhood education in providing sufficient opportunities for practice and skill development.^34^ While outdoor play is deeply embedded in the culture of Nordic countries and widely encouraged in early childhood education, in Portugal it is still perceived mainly as a break time rather than an integral part of learning and development.^35^ Furthermore, most outdoor spaces in Portuguese kindergartens lack essential features to provide preschoolers with meaningful play opportunities.^32^ These spaces often have reduced access to play resources, limited loose objects and materials, as well as a scarcity of natural areas and diverse surfaces.^35^ Another challenge is the predominantly sedentary nature of preschoolers’ PA levels, with only about one in three meeting the recommended PA guidelines^36^ despite outdoor spaces being organised mainly to promote PA and motor and active play,^35^ and despite Portugal’s favourable climate which allows children to be outdoors year-round.

Research has shown that outdoor play interventions improve preschoolers’ motor and socioemotional competences.^23 24^ These interventions can leverage environmental affordances in outdoor kindergarten settings to facilitate active play engagement, as they are well-suited to reach most children and provide an optimal opportunity to promote both PA and active play.^14^ In Portugal, research on intervention-based outdoor play programmes remains scarce, with most studies prioritising indoor activities. While some studies characterise outdoor play environments,^35 37^ randomised trials are limited, highlighting the need for high-quality, scalable interventions to foster PL.

To date, no studies in Portugal have examined the impact of active play on PL in outdoor settings, particularly concerning environmental affordances for exploratory movement experiences. As a result, the existing evidence is insufficient to support evidence-based decision-making for the development of effective programmes and policies.

To address these challenges, the PLAY UP intervention was designed as a co-created, affordance-based approach to foster PL in preschool children through active play in outdoor kindergarten settings. In this context, the present study aims to:

(1) evaluate the effectiveness of the PLAY UP intervention: a co-created, affordance-based approach combined with educator training, in promoting PL in preschool children; (2) examine its effects on PA, motor competence, executive functioning, and social, emotional, and behavioural competences, as well as to explore children’s movement patterns and interactions in outdoor environments and (3) assess the feasibility, acceptability and implementation of the intervention during the pilot phase.

Together, these findings may inform the design of evidence-based, scalable interventions and contribute to public health and educational strategies aimed at promoting PL from early childhood.

## Methods and analysis

### Study design

This study is a cluster-randomised trial, with randomisation at the kindergarten (KG) level, to be conducted in public preschools in Évora, Portugal. The trial includes three KG (clusters). One cluster (four classrooms) is being used in an initial pilot phase to assess feasibility, acceptability and implementation procedures. Following the pilot phase, the intervention will be implemented in the remaining two clusters (three classrooms each), with one assigned to the intervention condition and the other serving as a wait-list control group. The study follows a preintervention and postintervention assessment design. After the pilot phase, the two remaining KG will be randomised at the cluster level in a 1:1 ratio, with one allocated to the intervention group and the other to the wait-list control group. The random allocation sequence will be generated by an independent researcher not involved in recruitment, intervention delivery or outcome assessment, using a computer-generated randomisation procedure. Given the small number of clusters, simple randomisation will be used. The allocation will then be communicated to the research team responsible for implementing the intervention.

The initial pilot phase involves approximately 75 children from one KG, designed to assess the feasibility, acceptability and implementation procedures of the PLAY UP intervention.

The study commenced in January 2026 with recruitment and is currently ongoing, with completion scheduled for the end of June 2027. The intervention phase will consist of 15 sessions per classroom delivered over approximately 8–10 weeks, followed by postintervention assessments. The main comparative study phase will be implemented after completion of the pilot phase, following the same procedures.

### Setting

The study will be conducted in outdoor KG environments characteristic of public preschools in Évora. Each KG includes an outdoor play area comprising a combination of natural and built features, such as soil and concrete surfaces and fixed play equipment. These outdoor settings provide diverse environmental affordances that support varied forms of active play, movement and social interaction.

### Participants

Participants are preschool children aged 3–6 years enrolled in the participating public KG. A total of 218 children were identified as eligible for recruitment. Inclusion criteria were as follows: regular attendance in preschool and have no limitations to participate in intervention. Exclusion criteria were as follows: participating in a similar intervention programme for at least 3 months; having limitations (eg, physical limitations; medical conditions; neurodevelopmental conditions) to take the assessment tasks.

### Recruitment

Recruitment took place through informational sessions held during routine parent–educator meetings at the beginning of the second school term (January 2026). During these meetings, the research team presented the PLAY UP objectives, methodology and potential benefits. Parents received an information pack including the participant information sheet, informed consent form and the parent questionnaire to support informed participation.

Of the 218 potentially eligible children, two were excluded based on eligibility criteria and one declined participation, resulting in a final sample of 215 participants.

### Procedures

All assessments will be conducted after written informed consent has been obtained from parents or legal guardians. Children will be informed verbally about the study using age-appropriate language prior to participation.

Data collection will take place at two time points: baseline (preintervention) and postintervention, immediately after completion of the intervention.

Assessments will be conducted in the KG setting during school hours, either individually or in small groups, depending on the nature of the task. Each child assessment will last approximately 40 min. Data will be collected in familiar environments (eg, classroom, outdoor playground or quiet indoor spaces) to minimise disruption to daily routines.

A standardised assessment protocol has been developed to ensure consistency and reliability of data collection. All assessments will be administered by a trained research team with prior experience in evaluating young children, following standardised procedures.

A multimethod approach will be used, combining quantitative measures (questionnaires, standardised tasks, accelerometry, wearable sensors and Global Positioning System (GPS)-based positioning) and qualitative methods (eg, interviews). Validated instruments with established psychometric properties for Portuguese children will be used, in line with the study objectives.

Children will be assessed within the KG context using different modalities depending on the outcome measure (eg, outdoor environments for movement tracking and indoor spaces for cognitive or individual assessments).

Educators will complete questionnaires at school, while parents will complete questionnaires at home and return them to the research team.

### PLAY UP intervention

The PLAY UP intervention is a multidimensional, co-created programme designed to enhance children’s PL through active outdoor play and the intentional use of environmental affordances. The intervention is designed around two complementary and interrelated components. First, it adopts a child-centred co-creation approach, in which children’s perspectives, preferences and interactions with the outdoor environment are actively integrated into the design and adaptation of play activities. This approach is informed by data collected on children’s behaviours and experiences, allowing the intervention to be continuously shaped by their insights. By actively involving children in the co-creation process, the intervention aims to promote engagement, motivation, enjoyment and meaningful participation in active play. Second, the intervention includes a capacity-building component for educators, providing training and ongoing support to enhance their ability to facilitate outdoor active play and effectively leverage environmental affordances. This educator-focused approach is intended to strengthen implementation quality, while ensuring the sustainability and scalability of the intervention in real-world educational settings. Together, these two components ensure that the intervention is both informed by children’s perspectives and supported by educators’ practices, enhancing its relevance and applicability in real-world settings.

### PLAY UP intervention programme (children’s approach)

The PLAY UP intervention for children is based on a co-creation approach, in which children are actively involved in shaping the design and implementation of outdoor play activities. A central component of the intervention is the active involvement of children in the co-creation process, in line with the UNICEF^38^ initiative, ensuring their voices are heard and their rights upheld in decisions that affect them. Children’s perspectives, preferences and behaviours are continuously gathered through observation, participatory activities, and qualitative methods (eg, interviews). These insights are used to adapt activities, select materials, and reorganise play spaces, ensuring that the intervention remains meaningful, engaging, and contextually relevant. The intervention also incorporates data-informed decision-making, linking objective measures of children’s movement patterns (eg, spatial behaviour, PA levels and positional interactions) with qualitative insights. This allows the identification of frequently and infrequently used areas, preferred activities and patterns of social interaction, which inform the ongoing adaptation of the intervention. Sessions are designed to encourage diverse forms of active play, including locomotor, object-control and exploratory movements, as well as social and cooperative play. The use of flexible materials (eg, loose parts) supports creativity, autonomy and variability in movement experiences, enabling children to engage with the environment in multiple ways. The intervention aims to create rich outdoor play environments that promote PL by enhancing children’s motivation, engagement, confidence and competence in movement. Through this approach, children are positioned as active participants in their own learning process, contributing to the development of meaningful and developmentally appropriate play experiences.

The PLAY UP intervention is grounded in affordances theory, drawing on Gibson’s ecological approach^17 39^ to identify and characterise environmental features that provide opportunities for action in outdoor settings. Within an ecological dynamics’ perspective, the intervention aims to foster PL by prioritising, strengthening and expanding opportunities for children to actively engage with their environment.

Prior to the intervention, outdoor affordances for play, including physical, social and risk affordances, will be identified and categorised based on data collected using the Go-Exterior instrument and participatory methods (Photo Elicitation Interviews, PEI). This process enables the identification of environmental features that support active play and engagement, and risky play (eg, a flat open area affording running and jumping; a tree affording climbing and risk-taking).^14 40 41^

The intervention is developed and implemented through a co-creation approach, actively involving children in shaping play experiences. Children’s input, gathered through PEI and observational data, informs session design, selection of materials and equipment, identification of preferred and underused play areas, and recognition of PA versus sedentary behaviour (SB) zones, as well as social interaction spaces. This approach supports the prioritisation of meaningful play areas and encourages the exploration of less-used environments.

The research team comprises members with expertise in the design and implementation of interventions for preschool children in both indoor and outdoor contexts.^15 23 42^ Sessions will be delivered by a psychomotor therapist under weekly supervision by the principal investigator (PI), who has expertise in psychomotor practice and supervision.

The intervention consists of 15 outdoor sessions (60 min each), delivered twice weekly and integrated into the regular preschool routine involving the whole class. Sessions are led, as mentioned above, by a psychomotor therapist professional, with the classroom educator providing pedagogical support through a non-intrusive presence. This approach ensures safety, supports engagement in outdoor exploration, and promotes meaningful learning experiences.^13 15^ Each session follows a semistructured format,^15^ organised into three main components:

1. Experimentation time (warm-up; ~10 min)—children engage in free movement and spontaneous play, exploring the environment, materials and social interactions. This phase aims to awaken the body and attention, allowing children to perceive and actualise environmental and social affordances through autonomous and unstructured exploration.
2. Main section (~35 min)—this component is organised into three phases: (1) Reflective dialogue (~5 min): children reflect on their initial exploration, sharing experiences, preferred activities and newly discovered body movement possibilities. The adult facilitates reflection through open-ended questions, promoting awareness of actions, interactions and experiences. (2) Expanding possibilities (~25 min): children actively participate in decision-making, selecting activities and proposing ideas. The adult adopts a facilitative role, adapting activities to children’s interests while introducing new challenges and affordances (eg, exploring alternative ways of movement or using materials differently). This phase promotes creativity, motor exploration and social interaction. (3) Re-experimentation time (~5 min): children test and refine newly discovered movement strategies and affordances, engaging in freely chosen, unstructured individual and collective exploration.
3. Symbolisation and meaning-making (~15 min)—children reflect on their experiences through verbalisation and/or drawing. Guided by cognitive, emotional, descriptive and connective questions, this phase supports awareness of learning, emotions and the multiple ways spaces and materials can be used, fostering meaningful learning^13^.

The 15 sessions are organised into five progressive components (three sessions each), with increasing complexity as new skills and affordances emerge: (1) Exploring areas (sessions 1–3): informed by children’s input (PEIs); (2) Outdoor spaces and resources (sessions 4–6): informed by affordances identified using Go-Exterior; (3) objective data-driven (sessions 7–9): based on objective measures, including Spatial Activity Behaviour (SAB) and Child-Level Positional Interactions (CLPI), derived from accelerometers and GPS; (4) Introduction of loose parts (sessions 10–12): expanding play opportunities and social interaction (5) Introduction of portable equipment (sessions 13–15): enhancing physical and social affordances.

Sessions are conducted in outdoor KG environments, leveraging natural and built features that afford opportunities for movement and social interaction. To enrich play experiences, the environment will be supplemented with portable equipment (eg, balls, hoops), loose parts, and natural and recyclable materials (eg, cardboard boxes, tyres, paper tubes, textiles). These materials will remain available after the intervention to support continued engagement in outdoor active play. Activities are tailored to children’s developmental characteristics (eg, age, height, competencies) and informed by behavioural and contextual data, including CLPI, SAB and environmental assessments.

Given the outdoor nature of the intervention, contingency measures will be implemented to address weather-related challenges. To ensure continuity of sessions under adverse conditions (eg, rain or muddy environments), appropriate equipment (eg, waterproof boots, overalls and rain jackets) will be provided.

### PLAY UP intervention programme (educators’ approach)

Educators will participate in a capacity-building programme designed to support the implementation of the PLAY UP framework and the adoption of a co-creation approach to promote PL through active play and the use of outdoor environmental affordances. As key agents in early childhood development, educators play a central role in fostering and sustaining PL^3^, making their training essential for intervention effectiveness and long-term impact.

To enhance intervention quality and support professional development, a 25-hour certified training module, PLAY UP: Active Learning and Outdoor Play for Early Childhood Education, will be developed and accredited by the National Scientific-Pedagogical Council for Continuous Training. The programme comprises five workshops and contributes to educators’ continuous professional development and career progression. Participation may be certified (with assessment) or non-certified. Each workshop has a duration of 5 hours, delivered through a blended learning format, combining one face-to-face session (held at the university or KG) and one online synchronous session. The training follows an active learning approach, integrating practical activities, group reflection, video analysis and discussion of real-world scenarios to ensure meaningful engagement and applicability to practice. The workshops address key components of the PLAY UP framework, including (1) From theory to practice: integrating physically active play into preschool education; (2) Affordances in outdoor play: how space and materials shape movement, learning and development; (3) Children’s movement patterns and social engagement in KG settings; (4) Changing practices: co-creating and implementing PLAY UP in early childhood education and (5) Moving, exploring, creating: loose parts and PL for sustainable learning. A distinctive feature of the training is its context-sensitive approach, whereby workshop content is informed by data collected within each KG. This includes information on children’s movement behaviours, use of space and interaction patterns, allowing the training to be tailored to each educational context. Data-informed tools (eg, visual representations of movement patterns and space use) will be used to support reflection and guide pedagogical decision-making. Additional support will be provided through a dedicated online platform (Moodle) and the PLAY UP website, offering access to resources, activity guides and ongoing communication with the research team. Certified trainers from the research team, with multidisciplinary expertise and prior experience in early childhood education and intervention delivery, will lead all training sessions. In addition to formal training, educators will participate in the twice-weekly PLAY UP sessions delivered to children. This experiential component allows educators to observe, engage with and reflect on the intervention in practice, supporting the integration of PLAY UP principles into their daily routines.

### Outcomes and measures

Outcomes will be assessed using a multidimensional and multimethod approach, combining objective measures, standardised assessments and qualitative data, as summarised in table 1.

**Table 1:** Overview of outcomes and measures used in the PLAY UP study, including primary, secondary and process outcomes, corresponding instruments, respondents and assessment time points (baseline and postintervention)

Outcome category	Outcome	Measure/instrument	Respondent/source	Time point
Primary outcome	Motor competence	Movement Assessment Battery for Children–Second Edition	Child	T0, T1
Secondary outcomes	Social, emotional and behavioural competences	Preschool and Kindergarten Behaviour Scales	Educator	T0, T1
	Behavioural difficulties	Strengths and Difficulties Questionnaire	Parent	T0, T1
	Executive functioning	Head-Toes-Knees-Shoulders Task	Child	T0, T1
	Spatial Activity Behaviour	Accelerometry+GPS	Child	T0, T1
	Child-Level Positional Interactions	Sensor-based positional tracking	Child/group level	T0, T1
	Kindergarten Affordances for Physical Literacy	Go-Exterior + Photo Elicitation Interviews	Research team/educators/children	T0, T1
Process outcomes (pilot phase)	Feasibility	Recruitment, retention, participation, attendance, adherence	Children, educators, research team	Pilot phase
	Acceptability	Observational records and interviews	Children, educators, research team	Pilot phase
	Implementation quality	Dose, intensity, duration, fidelity, resources, barriers/facilitators and safety	Educators, research team	Pilot phase

GPS, Global Positioning System; T0, baseline/initial assessment; T1, postintervention assessment.

### Primary outcome measure

#### Motor competence

Motor competence, as an indicator of PL, will be assessed using the Movement Assessment Battery for Children–Second Edition (MABC-2), with the total standardised score used as the primary outcome. MABC-2^43^ is a standardised, norm-referenced instrument designed to evaluate motor performance in children through the execution of specific tasks under standardised conditions. For the present study, the appropriate age band for children aged 3 years 0 months to 6 years 11 months will be used. Within this age band, each child will perform eight tasks distributed across three domains: manual dexterity, aiming and catching and balance:

1. Manual dexterity skills—this domain includes three tasks: Coin insertion (MD1)—the child is asked to pick up 6 or 12 plastic coins from a table and insert them through a narrow slot into a plastic box. Bead threading (MD2)—the task consists of threading 6 or 12 plastic beads onto a string. Tracing path (MD3)—the child must trace a path between two lines without crossing the boundaries. For MD1 and MD2, performance is scored based on the shortest time taken to complete the task. For MD3, the score is based on the number of errors made while tracing the predefined path.
2. Aiming and catching—this domain includes two tasks: Catching a beanbag (A&C1)—the child is asked to catch a beanbag thrown from a distance of 1.8 m. Throwing a beanbag (A&C2)—the child must throw a beanbag onto a mat placed 1.8 m away. For both tasks, performance is scored based on the number of successful attempts out of 10 trials. For A&C1, the score reflects the number of successful catches, and for A&C2, the number of accurate throws landing on the target.
3. Balance skills—this domain includes three tasks: Single-leg balance (Bal1)—the child is asked to maintain balance on one leg for as long as possible, up to a maximum of 30 s. Toe-walking (Bal2)—the child must walk along a straight line (4.5 m) without allowing the raised heel to touch the ground. Performance is scored by the number of consecutive correct steps, up to a maximum of 15. Jumping on mats (Bal3)—the child must jump from a standing position with both feet together from mat to mat. The score corresponds to the number of consecutive successful jumps, up to a maximum of 5.

Raw scores obtained in each task are converted into age-standardised scores according to the norms provided in the MABC-2 manual. The MABC-2 total standardised score is derived from the manual dexterity, aiming and catching and balance domains.

### Secondary outcome measures

#### Social, Emotional and Behavioural Competences (educator-reported)

Children’s social-emotional skills and behavioural difficulties will be assessed using the Preschool and Kindergarten Behaviour Scale (PKBS; Portuguese version; ages 3–6 years;^44^). The Portuguese version of the PKBS-2 was adapted and validated by Gomes and Pereira^44^ and further supported by Merrell *et al*^45^. The PKBS is a teacher-report instrument completed by preschool educators to assess children’s social-emotional competencies and behavioural problems within the context of peer interactions, particularly during free play. It is designed for children aged 3 to 6 years. The scale comprises 67 items rated on a 4-point Likert scale ranging from 0 (never) to 3 (very often). The instrument is organised into two main scales: (1) the Social Skills Scale, consisting of 29 items divided into three dimensions—Social Cooperation, Social Interaction and Social Independence and (2) the Problem Behaviour Scale, comprising 38 items divided into two subscales—Externalising Behaviour Problems and Internalising Behaviour Problems. Scores are summed for each domain, with higher scores indicating stronger social skills or greater behavioural concerns. For the present study, both the PKBS Social Skills Scale score and the Problem Behaviour Scale score will be used as educator-reported outcomes, with higher scores indicating stronger social-emotional competence and greater behavioural concerns, respectively.

### Behavioural difficulties (parent-reported)

Children’s behavioural difficulties will be assessed using the Strengths and Difficulties Questionnaire (SDQ; Portuguese version; ages 4–17 years^46^). The SDQ is a brief parent-report psychosocial questionnaire consisting of 25 items designed to assess children’s strengths and difficulties, providing behavioural indicators of potential mental health problems. It includes five subscales: Emotional Symptoms, Conduct Problems, Hyperactivity/Inattention, Peer Relationship Problems and Prosocial Behaviour. Each item is rated on a three-point scale (0–2), and a total difficulties score is derived from the sum of the four problem subscales. Scores are classified as normal, borderline or clinically significant based on predefined cutoffs.

### Executive functioning

Key executive functions, namely inhibitory control, cognitive flexibility and working memory, will be assessed using the Head-Toes-Knees-Shoulders (HTKS) task^47^, which is appropriate for children aged 4 to 8 years. The task also requires sustained attention and is widely used as a measure of behavioural self-regulation. The HTKS is a structured self-regulation task in which the child is required to perform motor actions that are opposite to verbal instructions, thereby integrating multiple executive processes. Specifically, the child must (1) attend to the instructions, (2) use working memory to retain and apply task rules, (3) inhibit the natural motor response to the command and (4) demonstrate cognitive flexibility when task rules change. For example, when instructed to touch the feet, the child must instead touch the head, and vice versa; similarly, when instructed to touch the shoulders, the correct response is to touch the knees, and vice versa. The task is administered in three parts of increasing complexity, each consisting of 10 commands (feet/head; shoulders/knees; and a combined condition including all commands). Performance is scored as follows: two points are awarded for each correct response, one point for a self-corrected response and zero points for an incorrect response, resulting in a maximum score of 60 points. The total HTKS score will be used as a single composite score reflecting executive functioning, including inhibitory control, cognitive flexibility, working memory and sustained attention. Higher scores indicate better self-regulation, particularly in terms of inhibitory control.

### Spatial activity behaviour

SAB will be assessed by integrating accelerometry and GPS data, allowing the examination of the relationship between children’s PA, SB and their spatial location within the KG outdoor environment.

Accelerometry will be used to objectively quantify PA and SB levels, including time spent in light-to-vigorous PA and SBs. Children will wear ActiGraph GT3X accelerometers (ActiGraph, Pensacola, Florida, USA), with activity counts recorded in 15 s epochs.

Simultaneously, children’s spatial location during outdoor recess is tracked using QStarz BT-Q1000XT GPS devices, also recording data in 15 s intervals. This combined methodology enables the precise identification of where children engage in different activity intensities within the outdoor setting and allows the mapping of behaviour in relation to environmental features^48^.

Data are collected during outdoor recess periods (~30 min), during which children will wear both devices. This approach allows for the analysis of spatial patterns of PA and SB within the KG environment.

### Child-Level positional interactions

CLPI will be used to assess children’s collective movement behaviour in the KG playground and to examine how spatial positioning relates to peer interactions and social engagement. Real-time spatial positioning will be continuously tracked during outdoor recess (~30 min) using high-resolution sensor technology, enabling the capture of detailed movement trajectories and interpersonal positioning within the playground environment.^49^ From these data, interpersonal movement-related variables will be derived to characterise patterns of proximity, coordination and interaction between children, providing insights into the relationship between movement dynamics and social behaviour. Outcomes will include mean inter-child distance and frequency of proximity interactions during recess.

### KG affordances for physical literacy

The quality of the outdoor environment is assessed using the GO-Exterior instrument (original Portuguese version^50^), which is designed for application in early childhood education settings. The instrument involves direct observation of a group of children and the responsible educator in the outdoor space, typically over a period of approximately 60 min. GO-Exterior comprises two main components: (1) a structured observation grid and (2) a semistructured interview with the educator. The observation grid consists of 36 items organised into six analytical categories: (1) overall appearance and identity, (2) spatial dimension, (3) accessibility, (4) maintenance and safety, (5) action opportunities and (6) adult role and routines. Items are rated on a 3-point scale: 0 (‘not observed’), 1 (‘partially observed’) and 2 (‘observed’). In addition to quantitative scoring, each item includes a field for qualitative notes, allowing the observer to record contextual information that supports the assigned rating. The scoring system allows for the calculation of category-specific and overall scores, supporting the characterisation of the outdoor environment in terms of its quality, strengths and limitations. These data enable the identification of key areas for improvement and provide a structured basis for intervention planning.

To complement the observational data, a semistructured interview is conducted with the educator responsible for the observed group. This interview is carried out by the PI and aims to clarify and validate the observations, as well as to gather additional information regarding children’s use of the outdoor space, the role of adults in supporting outdoor play, family involvement and educators’ perspectives on outdoor learning. The information obtained through both components is integrated to develop a comprehensive profile of the outdoor environment. These data contribute to a more comprehensive understanding of how outdoor environments are used in practice and are used to support the interpretation of observational findings. In the context of the present study, the information gathered through the GO-Exterior instrument is also used to inform the design and refinement of the PLAY UP intervention.

PEI, following the approach proposed by Pyle,^51^ will be conducted to incorporate children’s perspectives into the research process and to support their active contribution to the intervention design. This method is particularly suitable for young children and is well established in early childhood research.

The interviews are conducted by the PI, a psychomotor therapist with extensive experience in child assessment. A photo elicitation technique will be used to explore children’s experiences in outdoor KG environments from their own perspectives. Prior to the interviews, children will take photographs in the outdoor space, capturing elements they perceive as meaningful for play and social interaction.

During the interviews, the child and the PI jointly examine and discuss these photographs, allowing the child to interpret and assign meaning to the images. This collaborative process facilitates child-centred data collection and enhances the depth of insights obtained.

The PI guides the conversation using open-ended questions focused on the child’s outdoor experiences, including where the child plays, the types of activities engaged in, interactions with peers and the environment, and perceptions of the outdoor space.

Particular attention will be given to: (1) preferences for physical environments that encourage or discourage engagement in both sedentary and physically active behaviours; (2) enjoyable experiences, sense of connection and appreciation of the environment in relation to movement and PA and (3) perceived physical and social affordances of the outdoor setting.

All interviews are audio-recorded and subsequently transcribed for analysis. Qualitative data will be analysed to generate insights that will inform the development and refinement of the PLAY UP intervention.

### Pilot phase: feasibility, acceptability and implementation quality

The study includes an initial pilot phase designed to assess the feasibility, acceptability and implementation quality of the PLAY UP intervention in outdoor KG settings.

This phase is being conducted in one KG (four classrooms), involving approximately 75 children, and will inform the refinement of the intervention prior to the main study phase.

Feasibility and acceptability will be assessed from multiple perspectives, including children, educators and the research team, using a combination of quantitative and qualitative methods.

Feasibility will be evaluated across several domains, including recruitment, retention and participation rates; access to participants and barriers to participation; attendance and adherence to intervention sessions; feasibility and suitability of assessment procedures and outcome measures; time and resources required to conduct assessments; adequacy of intervention dose, intensity and duration; and safety, including the monitoring and reporting of adverse events^52^. Acceptability will be assessed through indicators such as children’s engagement, participation and perceived enjoyment of the intervention, as well as educators’ and researchers’ perceptions of the intervention’s suitability and implementation. Additional qualitative data, including observational records and semistructured interviews with educators and children, will be collected to explore experiences, perceived benefits and potential areas for improvement.

Quantitative indicators (eg, recruitment, attendance and retention rates) will be analysed descriptively. Qualitative data will be analysed using thematic analysis, allowing the identification of key themes related to feasibility and acceptability.

This pilot phase corresponds to an exploratory, proof-of-concept stage, aimed at determining whether the intervention is feasible and acceptable. By addressing feasibility within a structured and evidence-based approach, this study will strengthen the intervention’s long-term impact, ensuring it remains accessible, adaptable and capable of promoting meaningful educational and developmental benefits.

### Sample size

The protocol includes an initial pilot phase involving approximately 75 children to assess feasibility, acceptability and implementation procedures.

For the main comparative phase, sample size was estimated using G*Power (F tests: analysis of variance repeated measures, within–between interaction). Assuming a moderate effect size (f=0.25), α=0.05, power=0.80, two groups, two measurement occasions (baseline and postintervention), a correlation among repeated measures of 0.50 and ε=1, the required total sample size was 34 children.

The overall recruited sample exceeds this individual-level requirement. Given the study design and the limited number of KG clusters in the comparative phase, findings related to intervention effects will be interpreted with caution, and effect estimates with CIs will be reported to support interpretation and inform future sample size assumptions.

### Data analysis

Data analysis will be conducted using an integrative mixed-methods approach, combining quantitative and qualitative data, and will be performed separately for the pilot phase and the main study phase.

For the pilot phase, feasibility and acceptability indicators will be analysed descriptively, including recruitment, retention and attendance rates, as well as indicators related to implementation, resources and safety. Preliminary changes between baseline and postintervention will be explored using within-group comparisons, as appropriate.

For the main study phase, quantitative outcomes will be analysed by comparing the intervention and wait-list control groups at baseline and postintervention. The choice of statistical tests will be guided by the distribution and characteristics of the data. Where assumptions for parametric analyses are not met, non-parametric tests will be used to compare baseline differences between groups and changes from baseline to postintervention between groups.

Effect sizes will be calculated and reported to support interpretation of findings. The specific metric used will depend on the statistical tests applied, and effect sizes will be interpreted according to established guidelines. Results will be presented as means and SDs or means and 95% CIs, as appropriate.

All statistical analyses will be conducted using a significance level of p<0.05.

Qualitative data, including interviews and observational data, will be analysed using thematic analysis, and findings will be integrated with quantitative results to support interpretation of feasibility, acceptability and intervention effects.

### Patient and public involvement

Children, educators and parents are actively involved in the PLAY UP project through a co-creation and multi-informant approach.

Children are engaged as active contributors in shaping the intervention. Through a co-creation process aligned with child participation principles, children’s perspectives and preferences inform the design of outdoor play sessions, including activity selection, use of space, materials, and social interaction areas. Children’s input is used to enhance motivation, engagement and enjoyment, and to prioritise meaningful play opportunities while encouraging exploration of less-used outdoor spaces.

Educators are involved as key partners in the intervention through pedagogical capacity-building and training, supporting both implementation and sustainability. Their perspectives are incorporated through active participation in the intervention process and through semistructured interviews used to contextualise observations and better understand children’s use of space, educator roles and family involvement.

Parents contribute to the study primarily through questionnaire-based input on children’s strengths, difficulties, and behavioural indicators, and through engagement activities embedded in routine parent–educator meetings. Informational sessions are used to communicate the study’s aims, procedures and potential benefits, and families are invited to participate in an outdoor play event designed to foster shared understanding and engagement with the intervention. The research team will organise an outdoor play-based event where parents and children can experience together some of the activities of the PLAY UP project. Patient and public involvement informed the design and feasibility of the intervention but did not influence the selection of outcomes or the statistical analysis plan.

### Ethics and dissemination

This study has been approved by the Human Research Ethics Committee of the University of Évora (Ref. #24101) and complies with the principles of the Declaration of Helsinki^53^ and all applicable national and European legislation, including the General Data Protection Regulation and guidelines issued by the Portuguese Data Protection Authority (Comissão Nacional de Proteção de Dados).

Approval to conduct the study was also obtained from the relevant school authorities, including school grouping headmasters, KG directors and classroom educators. Participation is entirely voluntary. Written informed consent is obtained from parents or legal guardians prior to participation. Children are informed about the study procedures using age-appropriate language and are explicitly informed of their right to withdraw at any time without consequences.

The study does not involve any foreseeable risks. To ensure confidentiality and data protection, all collected data are coded and analysed at the group level. Each participant is assigned a unique alphanumeric code; identifying information is stored separately in password-protected files accessible only to the PI. Electronic data are securely stored on password-protected institutional devices, and all procedures related to data collection, storage, processing, retention and destruction comply with ethical and data protection standards. Data will be retained for 5 years following study completion for publication, dissemination and verification purposes.

To ensure equity, children allocated to the wait-list control group will be offered the opportunity to participate in a similar intervention after completion of the study.

Study findings will be disseminated through open-access peer-reviewed publications, scientific conferences and academic presentations. Knowledge-transfer activities targeting educators and relevant stakeholders will be conducted using accessible formats. Dissemination will be carried out in collaboration with institutional communication offices and school partners, ensuring transparent, inclusive and ethically responsible communication.

Additional dissemination outputs will include workshops, seminars, newsletters, digital platforms and a freely available educational e-book describing the theoretical framework and intervention approach. All dissemination activities will comply with ethical standards and data protection regulations, focusing exclusively on aggregated data and avoiding identification of individual participants.

## Supplementary Material

Reviewer comments

Author's manuscript

## References

[1] Whitehead ME, Durden-Myers EJ, Pot N. The value of fostering physical literacy. J Teach Phys Educ 2018;37:252–61. 10.1123/jtpe.2018-0139

[2] Edwards LC, Bryant AS, Keegan RJ, et al. Foundations and Associations of Physical Literacy: A Systematic Review. 47. Sports Medicine, 2017:113–26. 10.1007/s40279-016-0560-7 PMC5215133 27365029

[3] Fröberg A, Lundvall S. Physical literacy assessment: a conceptualization and tools. In: Promot Phys Act Heal Sch Setting. 2024: 67–87.

[4] Jurbala P. What Is Physical Literacy, Really? Quest 2015;67:367–83. 10.1080/00336297.2015.1084341

[5] Young L, O’Connor J, Alfrey L. Physical literacy: a concept analysis. Sport Educ Soc 2020;25:946–59. 10.1080/13573322.2019.1677586

[6] Barnett LM, Mazzoli E, Hawkins M, et al. Development of a self-report scale to assess children’s perceived physical literacy. Phys Educ Sport Pedagogy 2022;27:91–116. 10.1080/17408989.2020.1849596

[7] Weir N, Pringle A, Roscoe CMP. Physical literacy and physical activity in early years education: what’s known, what’s done, and what’s needed? Children (Basel) 2024;11. 10.3390/children11111355 PMC11593175 39594930

[8] Truelove S, Vanderloo LM, Tucker P. Defining and measuring active play among young children: a systematic defining and measuring active play among young children: a systematic review. J Phys Act Health 2016;14:155–66. 10.1123/jpah.2016-0195 27775475

[9] Bento G, Dias G. The importance of outdoor play for young children’s healthy development. Porto Biomedical Journal 2017;2:157–60. 10.1016/j.pbj.2017.03.003 32258612 PMC6806863

[10] Nanda PD, Ndraha PS, Ginting S. The impact of outdoor play on motor skill development in early childhood: a systematic literature review. J Found Learn Child Dev 2025;1:141–56. 10.53905/ChildDev.v1i03.19

[11] Koepp AE, Gershoff ET, Castelli DM, et al. Preschoolers’ executive functions following indoor and outdoor free play. Trends Neurosci Educ 2022;28:100182. 10.1016/j.tine.2022.100182 35999013

[12] Brussoni M, Gibbons R, Gray C, et al. What is the relationship between risky outdoor play and health in children? a systematic review. Int J Environ Res Public Health 2015;12:6423–54. 10.3390/ijerph120606423 26062038 PMC4483710

[13] Bilton H. Outdoor learning in the early years. In: Management and innovation. Routledg, 2010.

[14] Almeida G, Guerreiro D, Pomar C, et al. Affordances” no espaço exterior: o exemplo do Programa Out to In. Cad Educ Infância 2021;123:10–5.

[15] Veiga G, Marmeleira J, Laranjo L, et al. Planning and developing outdoor-oriented practices with children. Universidade de Évora 2021;63–72.

[16] Veiga G, Marmeleira J, Laranjo L. The importance of outdoor practices for children’s health and development and for the comunity. In: Évora U de, ed. Taking the Best from outdoor play a pratical book for parents and practitioners of early childhood education. Évora: Veiga, G. Laranjo, L. Marmeleira, J. Almeida, G. Küçükturan, A. Évora: Veiga, 2021: 1–14.

[17] Gibson JJ. The Theory of Affordances. Hillsdale, NJ: Lawrence Erlbaum Associates, 1977.

[18] Bilton H. Physical Development and Outdoor Learning. A Practical Guide for the Early 810 Years. Routledg, 2026.

[19] O’Sullivan M, Davids K, Woods CT, et al. Conceptualizing physical literacy within an ecological dynamics framework. Quest 2020;72:448–62.

[20] Rietveld E, de Haan S, Denys D. Social affordances in context: what is it that we are bodily responsive to? Behav Brain Sci 2013;36. 10.1017/S0140525X12002038 23883765

[21] Borghi AM. Affordances, context and sociality. Synthese; 2018. Available: 10.1007/s11229-018-02044-1

[22] Bjørgen K. Children’s well-being and involvement in physically active outdoors play in a Norwegian kindergarten: playful sharing of physical experiences. Child Care in Practice 2015;21:305–23. 10.1080/13575279.2015.1051512

[23] Veiga G, Guerreiro D, Pomar C, et al. Effects of an outdoor play-based intervention on preschoolers’ fundamental movement skill competence: a cluster randomized controlled trial. Early Educ Dev 2025;36:1809–20. 10.1080/10409289.2025.2532220

[24] Veiga G, Guerreiro D, Marmeleira J, et al. OUT to IN: a body-oriented intervention program to promote preschoolers’ self-regulation and relationship skills in the outdoors. Front Psychol 2023;14. 10.3389/fpsyg.2023.1195305 PMC10435744 37599760

[25] Kiviranta L, Lindfors E, Rönkkö ML, et al. Outdoor learning in early childhood education: exploring benefits and challenges. Educ Res 2024;66:102–19. 10.1080/00131881.2023.2285762

[26] Hanscom A. Balanced and Barefoot: How Unrestricted Outdoor Play Makes for Strong, Confident, and Capable Children. New Harbinger Publications, 2016.

[27] James ME, de Lannoy L, Lopes O, et al. A systematic review and meta-analyses of the relationships between active outdoor play and 24-hour movement behaviors. J Sport Health Sci 2026;15:101115. 10.1016/j.jshs.2025.101115 PMC13053787 41475528

[28] Sherwin JC, Reacher MH, Keogh RH, et al. The association between time spent outdoors and myopia in children and adolescents: a systematic review and meta-analysis. Ophthalmology 2012;119:2141–51. 10.1016/j.ophtha.2012.04.020 22809757

[29] Fermin C, Perez M, Obee A, et al. Benefits of time spent outdoors in early childhood education: a systematic review. FIUURJ 2024;2:54–72. 10.25148/URJ.020107

[30] Senoz EG, Kol S. Playing outside, thriving inside: exploring the impact of outdoor play frequency on emotional intelligence in children aged 5–6. Eur Early Child Educ Res J 2025;33:287–301. 10.1080/1350293X.2024.2439949

[31] World Health Organisation (WHO). Literature review on chemical pollutants in indoor air in public settings for children and overview of their health effects. 2021.

[32] Almeida A, Rato V, Dabaja ZF. Outdoor activities and contact with nature in the Portuguese context: a comparative study between children’s and their parents’ experiences. Child Geogr 2023;21:108–22. 10.1080/14733285.2021.1998368

[33] EACEA / Eurydice. Key Data on Early Childhood Education and Care in Europe – 2025. Eurydice Report. Luxembourg: Publications Office of the European Union, 2025.

[34] Goodway J, Ozmun J, Gallahue D. Understanding Motor Development: Infants, Children, Adolescents, Adults. Burlington, MA: Jones & Barlett Learning, 2019.

[35] Bento G, Costa JA. Outdoor spaces design in early childhood settings – analysing opportunities for children’s play in Portugal. Int J Play 2022;11:270–88. 10.1080/21594937.2022.2101274

[36] Vale S, Mota J. Adherence to 24-hour movement guidelines among Portuguese preschool children: the prestyle study. J Sports Sci 2020;38:2149–54. 10.1080/02640414.2020.1775385 32516083

[37] Da Silva Pinto J. Brincar ao ar livre em educação de infância: possibilidades de participação das crianças. Rev Port Educ 2025;38. 10.21814/rpe.33384

[38] UNICEF. Child friendly cities. Available: http://childfriendlycities.org/ [Accessed 19 Feb 2025].

[39] Gibson JJ. The Ecological Approach to Visual Perception. Hillsdale, NJ: Lawrence Erlbaum Associates, 1979.

[40] Almeida G, Marmeleira J, Laranjo L, et al. Risky play in outdoor environments. In: Mendes R, Coelho-e-Silva MJ, Sá E, eds. Estudos de Desenvolvimento Motor da Criança XIII. Coimbra: Universidade de Coimbra, 2020: 63–5.

[41] Heft H. Affordances of children’s environments: A functional approach to environmental description. Child Environ Q 1988;5:29–37.

[42] Almeida G, Rasteiro A, Cruz-Ferreira A, et al. Efeitos de uma intervenção psicomotora combinando música e ritmo na coordenação motora de crianças em idade pré-escolar. In: Lagoa M, Coutinho D, Carvalho C, et al., eds. Estudos em Desenvolvimento motor da Criança XVI. Maia: Universidade da Maia, 2022: 211–6.

[43] Henderson S, Sugden D, Barnett A. Movement Assessment Battery for Children - 2. Pearson, Editor. London, 2007.

[44] Gomes RM, Pereira A. Análise confirmatória da escala comportamental para crianças do Pré-Escolar (PKBSpt): versão portuguesa || Confirmatory analysis of behavior scale for children in preschool (PKBSpt): Portuguese version. R Est Inv Psico y Educ 2018;5:92–101. 10.17979/reipe.2018.5.2.2914

[45] Merrell KW, Major S, Seabra-Santos MJ. Manual Da Versão Portuguesa Das Preschool and Kindergarten Behavior Scales – Second Edition. Hogrefe, 2021.

[46] Ferreira T, Geiser C, Cadima J, et al. The strengths and difficulties questionnaire: an examination of factorial, convergent, and discriminant validity using multitrait-multirater data. Psychol Assess 2021;33:45–59. 10.1037/pas0000961 33119377

[47] McClelland MM, Cameron CE, Duncan R, et al. Predictors of early growth in academic achievement: the head-toes-knees-shoulders task. Front Psychol 2014;5. 10.3389/fpsyg.2014.00599 PMC4060410 25071619

[48] Bai P, Schipperijn J, Rosenberg M, et al. Where are preschoolers active in childcare centers? A hot-spot analysis using GIS, GPS and accelerometry data. Child Geogr 2023;21:660–76. 10.1080/14733285.2022.2104627

[49] Gonçalves B, Esteves P, Folgado H, et al. Effects of pitch area-restrictions on tactical behavior, physical, and physiological performances in soccer large-sided games. J Strength Cond Res 2017;31:2398–408. 10.1519/JSC.0000000000001700 27806007

[50] Bento G. Grelha de Observação de Espaços Exteriores Em Educação de Infância: GO–Exterior. UA edições, 2000.

[51] Pyle A. Engaging young children in research through photo elicitation. Early Child Dev Care 2013;183:1544–58. 10.1080/03004430.2012.733944

[52] Abbott JH. The distinction between randomized clinical trials (RCTs) and preliminary feasibility and pilot studies: what they are and are not. J Orthop Sports Phys Ther 2014;44:555–8. 10.2519/jospt.2014.0110 25082389

[53] World Medical Association. World Medical Association Declaration of Helsinki: ethical principles for medical research involving human subjects. JAMA 2013;310:2191–4. 24141714 10.1001/jama.2013.281053

