# Supplementary material for: Physical Literacy through Active plaY and environmental affordances in outdoor kindergarten settings: a multidimensional approach to Unveiling Potential (PLAY UP) – study protocol for a cluster-randomised trial
Source: BMJ Open. 2026 Jul 1;16(7):e120976. doi: 10.1136/bmjopen-2026-120976 (PMC13358333; doi:10.1136/bmjopen-2026-120976)
Supplement: Reviewer comments [file bmjopen-2026-120976.reviewer_comments.pdf]

PEER REVIEW HISTORY

BMJ Open publishes all reviews undertaken for accepted manuscripts. Reviewers are asked to complete a checklist review form and are provided with free text boxes to elaborate on their assessment. These free text comments are reproduced below.

ARTICLE DETAILS

Title (Provisional)

Study protocol for a cluster-randomized trial fostering Physical Literacy through Active plaY and environmental affordances in outdoor kindergarten settings – a multidimensional approach to Unveiling Potential (PLAY UP)

Authors

Almeida , Gabriela; Gonçalves, Bruno; Luz, Carlos; Galhardas, Luís; Bilton, Helen; Marmeleira, José

This article was not externally reviewed at BMJ Open. Protocols that have been independently assessed prior to submission to BMJ Open are usually fast-tracked to publication on the grounds that further substantial changes will not be possible. This independent assessment will usually be external, independent review for both a substantial grant award from a non-commercial or government funder and ethics approval.
